# Advances in the Micropropagation and Genetic Transformation of *Agave* Species

**DOI:** 10.3390/plants11131757

**Published:** 2022-07-01

**Authors:** Erika Bautista-Montes, Laura Hernández-Soriano, June Simpson

**Affiliations:** Department of Genetic Engineering, Cinvestav Unidad Irapuato, Km 9.6 Libramiento Norte Carretera Irapuato-Leon, Guanajuato C.P. 36824, Mexico; erika.bautista@cinvestav.mx (E.B.-M.); laura.hernandez@cinvestav.mx (L.H.-S.)

**Keywords:** *Agave* genus, bulbils, co-cultivation, organogenesis, transformation rate

## Abstract

The *Agave* genus is composed of approximately 210 species distributed from south United States to Colombia and Venezuela. Numerous *Agave* species have been used for the preparation of alcoholic beverages and have attracted interest in the pharmaceutical and food industry. Despite their economic importance, there are few initiatives for the improvement and selection of characteristics of interest. This is mainly due to its morphology, long lifecycles, and monocarpic nature. Micropropagation is a feasible alternative to the improvement of *Agave* species. It has been used for multiple purposes, including massive propagation, induction of somaclonal variation to enhance agronomic characteristics of interest, maintenance of specific genotypes, and genetic transformation using molecular techniques. In this report, we summarize the most outstanding findings regarding the micropropagation of *Agave* species mediated by multiple regeneration responses. We also describe the media and growth regulators for each of the previously described methods. In addition, we discuss how micropropagation has allowed the development of transformation protocols. Exploitation of this technology may be a feasible strategy to introduce genes and improve certain traits. Genetic transformation also offers an opportunity for studying molecular mechanisms. This represents advantages for optimizing production in the field and for implementing breeding programs.

## 1. Introduction

### 1.1. Agave genus

*Agave* is an endemic genus from America, belonging to the subfamily Agavoideae [1]. It is composed of approximately 210 species (spp.), distributed from south United States to Colombia and Venezuela. Mexico is the main center of distribution of the genus, with 159 spp. in its territory, of which 119 spp. are endemic [2,3].

Agave plants are characterized by short stems with leaves organized in rosettes, with different levels of succulence, fibrous and with a terminal spine, their edges may have thorns [3]. The lifecycle in this genus varies among species, but normally it takes around 5 to 20 years before producing a paniculate or racemose inflorescence in the subgenus *Agave* and a spicate inflorescence in the *Littae* subgenus [4].

Bats and insects pollinate *Agave* in the wild (self and/or cross-pollination), forming capsules with black and triangular seeds, which are dispersed near the mother plant. Nevertheless, a low rate of germination of some species has been reported [5]. Another form of reproduction is through offsets from the rhizome and in rare cases bulbils from the inflorescence. Under cultivation, *A. tequilana*, offsets rather than seeds are used by producers to quickly obtain a large quantity of genetically identical plants [6].

### 1.2. Economic Importance of Agave Species

Since the pre-Columbian era, numerous *Agave* species have been used in daily life, for example for the preparation of alcoholic beverages, fibers, food, medicine, ornamental plants, etc. (Table 1). After the Spanish colonization of America, the tequila industry grew in importance since the Europeans brought the necessary technology for distillation [4]. However, several sources suggest that distillation technology was first introduced from the Philippines via the Pacific coast [7].

Nowadays, in Mexico a huge industry of tequila and mezcal exists which has grown internationally, since 2003 in the case of tequila, with the United States, Germany, and Spain as the major consumers. In the case of mezcal, most of the production is exported to the United States and Taiwan. According to the Tequila Regulatory Council, tequila production during 2021 was 527 million liters and foreign trade accounted for around USD 2355 million in 2020 [8,9]. It is estimated that tequila production will increase by around 27.13% within the next decade and mezcal production by 7.99% [10].

**Table 1:** Some uses of *Agave* species since pre-Hispanic times (modified from [11]).

Use	Part of the Plant	Species
Distilled beverages (mezcal)	Stems and leaves	*Agave americana* var. *americana*, *A. americana* var. *oaxacensis*, *A. angustifolia*, *A. asperrima*, *A. convallis*, *A. duranguensis*, *A. esperrimia*, *A. karwinskii*, *A. marmorata*, *A. palmeri*, *A. potatorum*, *A. rodacantha*, *A. salmiana*, *A. seemaniana*, *A.shrevei*, *A. tequilana* var. azul, *A. weberi*, A*. womomahi*, *A. zebra*
Fermented beverages (Aguamiel and pulque)	Stems and leaves	*A. americana* var. *americana*, *A. angustifolia*, *A. atrovirens*, *A. gracillispina*, *A. hookeri*, *A. macroculmis*, *A. malliflua*, *A. mapisaga*, *A. salmiana* var. salmiana, *A. tecta*
Fibers	Leaves	*A. americana* var. *americana*, *A. americana* var. *oaxacensis*, *A. angustifolia* var. *angustifolia*, *A. convallis*, *A. fourcroydes*, *A. ghiesbreghtii*, *A. horrida*, *A. lechuguilla*, *A. sisalana*
Food	Stems, leaves, flower stalk, flowers	*A. americana*, *A. angustiarum*, *A. angustifolia*, *A. applanata*, *A. chiapensis*, *A. karwinskii*, *A. marmorata*, *A. potatorum*
Medicinal	Leaves, cuticle, juice	*A. americana*, *A. angustiarum*, *A. marmorata*, *A. potatorum*, *A. seemanniana*
Ornamental	Whole plant	*A. americana* “Marginata”, *A. applanata*, *A. dasylirioides*, *A. desmettiana*, *A. guiengola*, *A. isthmensis*, *A. macrocacantha*, *A. salmiana*, *A*. *stricta*

In addition to fermented and distilled beverages, the *Agave* genus has attracted interest in several other areas of application in recent decades. The exploitation of the bioactive compounds of *Agave* has been considered by the pharmaceutical industry for use as auxiliaries due to their various biological effects, such as antimicrobial, antifungal, antioxidant, anti-inflammatory, antiparasitic, anticancer, and immunomodulatory activity [12]. On the other hand, agave carbohydrates have been used as substitutes for sugars and fats with many applications in the food industry [13]. Potential use of agave species in bioethanol production is also promising due to significant biomass production, CAM physiology and the ability to survive in arid climates, leading to lower environmental impacts during production in comparison to sugarcane molasses or other sources of bioethanol [14,15,16].

### 1.3. Genetic Improvement of Agave Species

Despite the economic importance of several *Agave* species as crops, there are few initiatives for the improvement and selection of characteristics of interest. The lack of breeding programs in *Agave* is mainly due to the morphology of the plants, their long lifecycles, and their monocarpic nature [17]. Hence, it is essential to develop alternative strategies that contribute to the improvement of *Agave* species.

Breeding programs through crosses have been limited. The most outstanding has been Hybrid H11648, which is a cross between *Agave amaniensis* X *Agave angustifolia* and backcrossed with *A. amaniensis*. It was released during the early 20th century in Tanzania with the aim of producing higher fiber yields in a wide variety of climatic conditions and soil types [18]. To date Hybrid H11648 is the cultivar of choice for fiber production globally, including in China and Brazil where most agave fiber is produced [19].

From 1993 to 2006, an agave genetic improvement program for fiber production was developed in Cuba at the “Liliana Dimitrova” Horticultural Research Institute. It consisted of the development of agave accessions from five mother plants selected in henequen production fields with few thorns at the edges of the leaves. In addition, accessions were developed from the segregation of *Agave fourcroydes*, and some other accessions were developed from Hybrid H11648. Accessions 97 and “Liliana CH” were the most outstanding, showing greater vegetative development and greater amounts of dry fiber, respectively [20].

Likewise, intraspecies and interspecies crosses have shown greater seed production and viability in *A. tequilana* and *A. americana*. Based on this, crosses between different *A. tequilana* cultivars could improve the level of fertility and maintain broader germplasm [5]. However, the Official Mexican Standard NOM-006-SCFI-2012 restricts tequila production to the use of at least 51% of total reducing sugars derived from *A. tequilana* var. azul and no other variety or cultivar to produce tequila [21]. Even somaclonal variants of *A. tequilana* var. azul, such as the “manso” phenotype characterized by leaves without thorns on the edges, are prohibited under the strict classification [22]. These restrictions are a further drawback to the implementation of breeding programs for *Agave* species that are exploited for the production of alcoholic beverages.

## 2. Micropropagation in Agave Species

An alternative to conventional propagation in *Agave* is the use of plant tissue and cell cultures, in order to easily obtain new plantlets in a short time and on a large scale. Micropropagation presents several advantages which are directly applicable for commercial Agave production, including mass production of plantlets within a short timescale; micropropagated plants, which are free from pathogens thus reducing the spread of diseases between plantations; and the production of plants that are uniform in age and size, leading to homogeneous plantations which facilitate and optimize the process of harvesting. Consequently, several of the main tequila companies are currently testing the wide-scale use of micropropagated *A. tequilana* germplasm in comparison to the traditional practice of planting offsets [23].

Since the 1980s, several species have been propagated using micropropagation for multiple purposes, including massive propagation of endangered species such as *A. arizonica* and *A. victoria-reginae*; induction of somaclonal variation to enhance agronomic characteristics of interest; maintenance of specific genotypes, as in the case of the tequila industry; and genetic transformation using molecular techniques [24,25,26,27,28].

**Figure 1:**
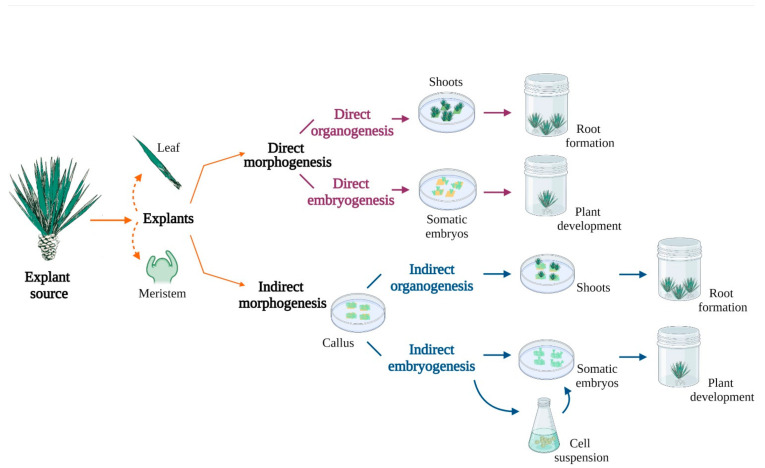
Regeneration responses in Agave species. Regeneration can be induced from explants, as leaf and meristem tissues. This has been carried out through different approaches such as direct organogenesis, allowing the development of shoots with no intervening callus; and indirect organogenesis, which is the formation of callus before the development of shoots. Direct and indirect somatic embryogenesis are also mainly used and consist of the directly formation of embryos from a cell or small group of cells, and the production of callus from the explant before the production of embryos respectively [29]. Image created with https://app.biorender.com (accessed on 14 February 2022).

Regeneration responses have been achieved through various approaches (Figure 1) including axillary bud proliferation, as reported by Ramírez-Malagón, for *A. tequilana*, *A. salmiana* ssp. *Crassispina*, *A. duranguensis*, *A. oscura*, *A. pigmaea*, and *A. victoria-reginae*. For *A. tequilana*, regeneration was obtained through temporary pulse treatments with different concentrations of 2,4-D, yielding 12 shoots per explant with 6.8 mM 2,4-D and three days of exposure to the hormone. Regarding the other species, the pulse system did not achieve any shoot formation since explants became necrotic. Nevertheless, IBA and BA were also tested at different concentrations, in the other species leading to axillary shoot formation as expected [29,30].

Direct organogenesis is another response exploited for micropropagation and there are several reports where new shoots have been induced. For instance, in *A. sisalana*, *A. fourcroydes*, and *A. cantala*, several combinations of growth regulators were evaluated, where 0.40 µM NAA + 0.49 µM IBA + 2.32 µM KIN proved to be the best combination to promote shoot formation and avoid callus production during the process [30].

On the other hand, indirect organogenesis is also a way to induce shoots. In this case it is necessary to first pass through a callus induction phase and then promote shoot proliferation. It has been observed that undifferentiated tissues, such as meristem cells, are an appropriate choice to induce new buds, even for direct somatic embryogenesis [31]. Indirect organogenesis in *A. tequilana* was reported, obtaining a suitable response when meristems were exposed to different zeatin and 2,4-D combinations to produce callus, which was grown on in 5.2 µM NAA in order to maintain indefinitely the growth of callus tissue. For promoting shoot formation, the treatment that showed the best results was 0.11 µM 2,4-D in combination with 44 µM BA, with a bud forming capacity (BFC) index of 14.5. This BFC index represents the relation between the mean number of buds per explant and the percentage of explants forming buds [32].

Other systems of regeneration widely used in many plant species are direct and indirect somatic embryogenesis, considered a viable alternative for genetic improvement, since cultures initiate from a single cell or, in some cases, a group of cells. Rodríguez-Garay reported for the first time the production of somatic embryos in the genus *Agave*, using 2,4-D as a growth regulator. Somatic embryos were produced on leaf blades of in vitro *A. victoria-reginae* plantlets when the medium was supplemented with 1.4 µM 2,4-D and germination was achieved on half-strength MS medium without growth regulators [25].

An example of indirect somatic embryogenesis is that reported by Tejavathi. The response was obtained in *A. vera-cruz Mill*, the main source of natural fiber in India. 2,4-D and NAA were shown to successfully produce embryogenic callus, in comparison to IAA and IBA, which produced non-embryogenic callus. The addition of 5.37 µM NAA + 0.91 µM Zeatin + 40 g/L sucrose to the medium was the best combination for somatic embryogenesis in this species [33].

Independently of the regeneration response, micropropagation may also be combined with various methodologies for mass propagation. The most common system is semisolid culture, although there are additional techniques, such as temporal immersion systems and thin cell suspension layers, that are suitable options for enhancing the number and quality of shoots/somatic embryos obtained [34,35]. Table 2 summarizes in detail research on *Agave* micropropagation, focusing on reports since 2000 onwards.

### 2.1. Medium, Growth Regulators and Response Comparison of Agave Species Micropropagation

Depending on the intended use, micropropagation protocol development for *Agave* species is focused on specific regenerative pathways, as mentioned above, and the use of specific growth regulators also corresponds to the expected response. Moreover, it is important consider that results may vary within the same genus under similar cultivation conditions [29] and even different genotypes of the same species may influence the responses obtained. Therefore, it is fundamental to establish a general micropropagation protocol that will serve for the majority of *Agave* species. 

In many reports for *Agave* species, authors used MS salts, supplemented with L2 vitamins, and in some cases, modified the ammonia concentration. A common growth regulator used to produce an indirect response, as in the case of organogenesis or somatic embryogenesis, is 2,4-D at a concentration ranging from 0.1 to 9.05 µM. However, it has also been reported to directly promote shoot formation in combination with cytokinins such as BA or KIN, with concentrations ranging from 4.44 to 38.2 µM and 2.32 to 27.84 µM, respectively (Table 2). 

Another example is the case of *A. cantala*, *A. fourcroydes*, *A. sisalana*, and *A. peacockii*. Contrasting concentrations of KIN are required to induce direct organogenesis: 27.84 µM for *A. peacockii*, and 2.32 µM KIN for the others. In addition, *A. peacockii* also requires the supplement of the cytokinin BA, whereas the other species require the addition of the auxins NAA and IBA. The reported numbers of shoots generated under these conditions vary extensively from 87 to 4, respectively [30,44] 

Regarding regeneration efficiency, the system with the highest regeneration rate for the *Agave* genus is that of somatic embryogenesis, both direct and indirect. Table 2 shows the formation up to 556 somatic embryos for *A. tequilana* [35], by organogenesis, the number of regenerated shoots was up to 87 in *A. peacockii* [43]. Nonetheless, when somatic embryogenesis is carried out, more time is needed to achieve shoot regeneration, and a balance must be reached for each specific project in relation to timeframe and funding.

### 2.2. Genetic Transformation in Agave Species

The development of micropropagation methods represents a significant opportunity to develop genetic transformation protocols. Exploitation of this technology may be a feasible strategy to introduce genes and improve certain traits, such as tolerance to diseases, and increase the production of probiotic compounds.

During the last two decades, protocols to transform *Agave* species have been tested with promising, although variable, results (Table 3). For example, a patent for genetic transformation of the *Agave* genus by particle bombardment involving *A. tumefaciens* and *A. rhizogenes* has been filed. In the bioballistic method, embryogenic calli were bombarded with tungsten particles covered with plasmid DNA containing marker genes. On the other hand, embryogenic calli were placed in co-culture with *Agrobacterium* for 48 h and then transferred to a selective medium to obtain transformed cells. *PPT/Bar* and *hpt* genes (which confer resistance to phosphinothricin and hygromycin respectively) were used as selectable markers and the *uidA* gene (β-glucuronidase) was used as a reporter gene. Herbicide- or antibiotic-resistant plants were obtained using these protocols [45].

In a subsequent report, *A. salmiana* was transformed using co-cultivation with *A. tumefaciens* and particle bombardment. The *uidA* gene was used as a reporter gene in both cases, and *nptII* (neomycin phosphotransferase II) and *bar* genes were used as selectable markers for *A. tumefaciens* or bioballistic mediated transformation methods, respectively. The conditions for both shoot regeneration and rooting were optimized using leaves and embryogenic calli. *Agrobacterium* co-cultivation was the most effective method, obtaining 32 rooted transgenic plants regenerated from calli, with a transformation efficiency of 2.7%. The transgenes were detected in 11-month-old plants. Alternatively, the particle bombardment protocol produced transgenic calli that tested positive with the GUS assay after 14 months on a selective medium [28].

Likewise, root regeneration was induced in leaves, stems, and roots of *A. salmiana* mediated by *A. rhizogenes* A4. In vitro plantlets were inoculated with different concentrations of bacteria and acetosyringone. Leaf tissue showed the best response, producing 63% of transformed roots when 1 × 10^9^ bacteria mL^−1^ and 200 µM acetosyringone were used. The *nptII* and *uidA* genes were used as a selectable marker and a reporter gene, respectively. A rate of transformation of 80% of the tissues was determined for the reporter gene and 60% for the selectable marker [46]. 

A successful example of the use of transgenic agave plants is in the case of zebra disease, which is caused by *Phytophtora nicotianae* and attacks Hybrid 11648 in all regions where it is cultivated. Conventional plant breeding could be a strategy for obtaining plants tolerant to zebra disease. However, this method is difficult to achieve due to the long lifecycle of the hybrid, which takes around 10 years to bloom. Hence, a transgenic strategy could be an alternative to produce enhanced tolerance to *P. nicotianae* in Hybrid 11648 plants in a short period. Therefore, a transgenic strategy to express hevein-like peptides in calli of Hybrid 11648 was reported. The optimum culture media for callus induction were SH, 13.2 µM BA, 2.68 µM NAA, and 0.45 µM 2,4-D. The shoot regeneration media were SH, 6.66 µM BA, and 2.68 µM NAA. Several factors influencing transformation efficiency were also tested. The effective time for infection was 10 min and acetosyringone was used at a concentration of 200 µM. The optimum time for pre-culture of callus was three days, and the optimum co-culture time was four days. Thirty-seven lines from 150 explants were obtained and the hevein-like gene was expressed in seven lines [47].

**Figure 2:**
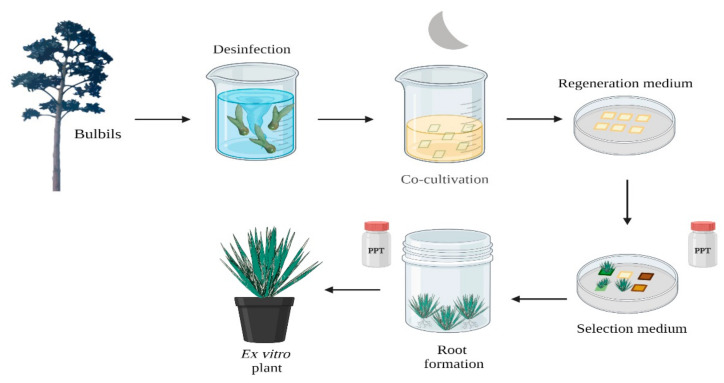
Genetic transformation Protocol. A method for the transformation of *A. desmettiana* bulbils was established mediated by *A. tumefaciens.* Shoots were developed by direct organogenesis [48]. Image created with https://app.biorender.com (accessed on 14 February 2022).

In spite of these successful reports of transformation of *Agave* species, the process is laborious and time consuming and development of a rapid and easy transformation protocol would be a great advantage. A method for the transformation of *A. tequilana* and *A. desmettiana* mediated by *A. tumefaciens* was therefore developed based on direct organogenesis. Bulbil meristems were used as explants and co-cultivated with *A. tumefaciens* strains LBA4404 and GV2260 using phosphinothricin (PPT/Bar) as the selective agent (Figure 2). *A. desmettiana* produced a much higher number of shoots per explant in comparison with *A. tequilana* (2–20 shoots and 1–2 shoots respectively) [48].

## 3. Conclusions and Prospects

The potential for exploitation of agave plants in applications other than the production of alcoholic beverages is being slowly recognized. In particular, the use of *Agave* species is gaining more relevance currently, due to applications related to tolerance to arid climates, bioenergy production, and the production of inulin (agavin). On the other hand, the growing success of the tequila and mezcal industries requires strategies for enhancing the agronomic characteristics of these plants.

Despite the difficulties involved in traditional breeding, many plant tissue culture protocols have been published over the years, with different regenerative responses. These protocols are the background for developing genetic transformation protocols. Recently, reports on *A. tequilana*, *A. salmiana*, and Hybrid 11648 have described promising results regarding the expression of antibiotic resistance genes and tolerance to zebra disease.

Thus, it is necessary to improve these protocols and explore alternative methodologies for propagation and genetic transformation to compare the transformation efficiency and costs. A suitable option for micropropagation is through direct organogenesis using the principal meristem from bulbils or offsets, since this is a source of pathogen-free explants and because of the relatively short time needed obtain new shoots. Explants are easy to obtain and process, and in addition fewer tissue culture steps are needed. In the case of genetic transformation, *A. tumefasciens*-mediated transformation is cheaper and more straight forward, with no requirement for special equipment or modified treatments during cultivation in vitro, as is the case with the bioballistic method.

Expense must be considered in any protocol development, especially if it will be used on an industrial scale. Additionally, it is indispensable to apply these protocols in other *Agave* species of potential interest.

Besides the improvement of certain agronomic characteristics, genetic transformation offers an opportunity for studying molecular mechanisms, such as the vegetative-to-reproductive transition (an important trait for commercial agave production) and fructan production in *A. tequilana.* Development of efficient strategies for propagation present several advantages for optimizing production in the field and implementing coordinated breeding programs.

Owing to the production of distilled beverages and traditional uses of agave species in Mexico, there is great potential for progress in this field. However, it is necessary to consider the restrictions for the use of genetically modified organisms and especially in the case of native species such as *Agave* or maize that are associated with ancient cultural traditions. The advent of more precise methods of genetic modification based on CRISPR-CAS technology could provide an avenue for unlocking the enormous potential of *Agave* species for many different applications [49].

## Figures and Tables

**Table 2:** Micropropagation protocols for *Agave* species.

Species	Source of Explant	Culture Media and Growth Regulators	Response	Rate	References
*A. fourcroydes*	Rhizome and stem tissue	Callus induction: Gamborg 1.12 µM 2,4-D + 4.44 µM BA Shoot regeneration: MMS 1.12 µM 2,4-D + 4.44 µM BA	IO	Abundant rootlike structures. 4 shoots per callus after 12 to 16 weeks of subculture	[26]
*A. arizonica*	Bulbils	Callus induction: MMS 1.4µM 2,4-D Shoot regeneration: MMS 44.4µM BA + 0.5 or 5.4 µM NAA	IO	5–10 shoots per callus after 12 weeks	[24]
*A. sisalana*, *A. fourcroydes*, *A. cantala*	Stolons	MS 0.40 µM NAA + 0.49 µM IBA + 2.32 µM KIN	DO	Multiplication index ranged between 3 and 4 for 4–5 weeks of subculture	[30]
*A. victoria-reginae Moore*	Young leaf blades from in vitro plantlets	Induction of somatic embryogenesis: MS medium + L2 vitamins + 1.4 µM 2,4-D Germination: half-strength MS without growth regulators	DSE	Not specified, but there were somatic embryos at the globular stage on 100% of the explants after 2 weeks	[25]
*A. parrasana*	Offshoots multiplied in vitro	MS + L2 vitamins + 13.3 µM BA	DO	19.9 shoots per explant	[36]
*A. sisalana*	In vitro grown immature leaves and rhizome	Callus initiation: MS + 9.05 µM 2,4-D + 4.6 µM KIN (In light conditions) Shoot regeneration: MS + 26.6 µM BA	IO	25.3 shoots per callus after 21 days of co-culture.	[37]
*A. vera-cruz Mill*	Shoot apices; cotyledons and leaf segments; and seeds	Induction of somatic embryogenesis: MS + L2 vitamins + 4.52 μM 2,4-D or 5.37 μM NAA, 4.8 μM IBA and 5.70 μM IAA Maturation: 5.37 μM NAA + 0.91 μM zeatin	ISE	Shoot tip: 16 embryos per explant Cotyledon: 17 embryos per explant In vitro leaf: 17 embryos per explant	[33]
*A. grijalvensis*	Germinated seeds in vitro	Shoot induction: MS + 38.2 µM BA	DO	Not specified	[38]
*A. tequilana*	Offsets	MMS + 6.8 mM 2,4-D for 3 days,	DO	12 axillary shoots per explant after 60 days	[29]
*A. salmiana* ssp*. crassispina*,	Offsets	0.49 µM IBA + 4.44 µM BA	DO	3 axillary shoots per explant after 60 days	[29]
*A. duranguensis*	Offsets	0.049 µM IBA + 4.44 µM BA	DO	6 axillary shoots per explant after 60 days	[29]
*A. oscura*	Offsets	2.46 µM IBA + 4.44 µM BA	DO	13 axillary shoots per explant after 60 days	[29]
*A. pigmaea*	Offsets	0.49 µM IBA + 13.31 µM BA	DO	6 axillary shoots per explant after 60 days	[29]
*A. victoria-reginae*	Offsets	2.46 µM IBA + 2.22 µM BA	DO	6 axillary shoots per explant after 60 days	[29]
*A. tequilana* var. azul	Callus from in vitro plants	9 µM 2,4-D + 1.3 µM BA	ISE	556.8 embryos using a celular suspension 161 × 10^3^ cell mL^−1^	[35]
*Hybrid N11648*	Shoot tip and immature leaf tissues	Callus induction: MS + 8.88 µM BA + 1.07 µM NAA Shoot regeneration: SH medium + 22.2 µM BA + 0.1 mg/l NAA + 0.492 µM IBA	IO	Shoot tip: 13.9 shoots	[39,40]
*A. fourcroydes*	In vitro plantlets	Induction of somatic embryogenesis: MS + L2 vitamins + 2.26 µM dicamba or 2.07 µM picloram Germination: half-strength MS without growth regulators	DSE	92.22 embryos/explant, and 81.72 embryos/explant respectively	[31]
*A. salmiana*	Plantlets from in vitro germinated seeds	MS + L2 vitamins + 0.18 µM 2,4-D + 44.4 µM BA	DO	14 axillary shoots	[40]
*A. americana*	Meristematic tissue	Callus induction: MS + 0.11 µM 2,4-D + 58.7 or 73.3 µM BA Shoot regeneration: MS medium without growth regulators	IO	71 shoots per callus after 36 weeks	[41]
*A. americana*	Shoots extracted from rhizomes	Embryo induction: MS + L2 vitamins + 10.3 µM picloram Callus induction 9.04 µM 2,4-D Germination: MSB medium without growth regulators	ISE	Not specified	[42]
*A. peacockii*	Rhizomatous shoots	MS + 26.6µM BA + 27.84 µM KIN	DO	87 shoots after 60 days of co-culture	[43]
*A. angustifolia*	In vitro plants	MMS 0.1 µM 2,4-D + 44.4 µM BA SS: MS + 0.1 µM 2,4-D + 44.4 µM BA TIS: RITA bioreactor	DO	6.23 shoots per plant	[34]
*A. guiengola*	Axillary sprouting from stem segments	MS + 8.88 µM BA	DO	3.7 shoots per explant	[44]

2,4-D: 2,4-dichlorophenoxyacetic acid, BA: 6-benzylaminopurine, IAA: Indole-3-acetic acid, IBA: indole-3-butyric acid, KIN: kinetin, NAA: α-naphthaleneacetic acid, MS Murashige and Skoog, MMS modified Murashige and Skoog, SH: Schenk and Hildebrandt medium, DO: Direct Organogenesis, DSE: Direct Somatic Embryogenesis, IO: Indirect Organogenesis, ISE: Indirect Somatic Embryogenesis. TIS: Temporary Immersion System.

**Table 3:** Genetic transformation protocols for *Agave* species.

Species	Method	Selectable Marker and Reporter Gene	Culture Conditions	Rate	Reference
*Agave* genus	Co-cultivation with *A. tumefaciens*	*bar;* *uidA*	Information not specified	Resistant plants to herbicide or antibiotic	[45]
	Particle borbadment	*hpt;* *uidA*		Resistant plants to antibiotic	
*A*. *salmiana*	Co-cultivation with *A. tumefaciens*	*nptII;* *uidA*	Bacteria exposure: 30 min Co-cultivation: MS50 + 5 µM BA + 2.7 µM NAA + 100 µM acetosyringone Callus induction: MS50 + 5 µM BA + 2.7 µM NAA + 10 mL L^−1^ cocktail 20 + 50 mg L^−1^ Kn + 250 mg L^−1^ Cf Rooted media: MS50 + 1.14 µM IAA + 12.5 µM BA + 50 mg L^−1^ Kn	32 rooted transgenic plants; transgenic calli	[28]
	Particle bombardment	*bar*, *uidA*	Callus induction: MS + 5 µM BA + 2.7 µM NAA Somatic embryos induction: MS + 5 µM BA + 2.7 µM NAA + 10 mL L^−1^ cocktail 20 Bombard pressure 1100 psi Selection media: MS + 5 µM BA + 2.7 µM NAA + 10 mL L^−1^ cocktail 20 + 0.5 mg L^−1^ BASTA	Green calli	
*A*. *salmiana*	Co-cultivation with *A*. *rhizogenes*	*nptII*, *uidA*	Mechanical injury with a needle + 1 × 10^9^ bacteria mL^−1^ + 200 µM acetosyringone Co-cultivation 6 days with no light Response induction: MS + 500 mg L^−1^ Cf	Transformed roots	[46]
Hybrid 11648	Co-cultivation with *A*. *tumefaciens*	*bar*, *uidA*	Callus induction: SH + 13.2 µM BA + 2.68 µM NAA + 0.45 µM 2,4-D + 6.5 g L^−1^ carrageenan Induction medium: SH + 6.66 µM BA + 2.68 µM NAA + 6.5 g L^−1^ carrageenan + 2 mg L^−1^ PPT Rooting induction: SH IAA	37 Transformed lines	[47]
*A*. *tequilana* and *A*. *desmettiana*	Co-cultivation with *A*. *tumefaciens*	*bar*, *uidA*	Strain: GV2260 and LBA4404 Shoot induction: BA + IBA, concentrations not especified	Shoots, transformation not specified	[48]

*bar* gene: resistance to phosphonithricin, *hpt*: resistance to hygromycin, *nptII*: resistance to neomycin and kanamycin, *uidA*: encodes the beta-glucuronidase enzyme. 2,4-D: 2,4-dichlorophenoxyacetic acid, BA: 6-benzylaminopurine, IAA: Indole-3-acetic acid, IBA: indole-3-butyric acid, KIN: kinetin, NAA: α-naphthaleneacetic acid, MS: Murashige and Skoog, MS50: half-strength Murashige and Skoog, SH: Schenk and Hildebrandt, Kn: kanamycin, Cf: cefotaxime, PPT: phosphinothricin.

## Data Availability

Data sharing does not apply to this article as no datasets were generated or analyzed during the current study.
